# Meta-analysis of mortality-associated factors in primary Sjögren’s syndrome patients with interstitial lung disease

**DOI:** 10.1007/s10067-024-07191-0

**Published:** 2024-10-21

**Authors:** Ruochen Pang, Xiaopeng Ma, Huifang Guo, Xuan Qi

**Affiliations:** 1 The Second Hospital of Hebei Medical University, No. 215, Heping West Road, Xinhua District, Shijiazhuang, 050000 Hebei Province China; 2 Department of Rheumatology and Immunology, the Second Hospital of Hebei Medical University, No. 215, Heping West Road, Xinhua District, Shijiazhuang, 050000 Hebei Province China

**Keywords:** Interstitial, Lung diseases, Meta-analysis, Prevalence, Primary Sjögren’s syndrome, Prognosis

## Abstract

The study aims to conduct a meta-analysis on 5-year survival rate and mortality-related factors in the patients with primary Sjögren’s syndrome concomitant with interstitial lung disease (pSS-ILD). Following the PRISMA (Preferred Reporting Items for Systematic Reviews and Meta-Analyses) guidelines, we searched various platforms and databases until November 22, 2023. We used the Newcastle–Ottawa Scale (NOS) for quality assessment and extracted study characteristics and effect sizes. The pooled 5-year survival rate, hazard ratios (HRs), and the corresponding 95% confidence intervals (95% CIs) were then calculated. A p-value of less than 0.05 was considered statistically significant. Patients with pSS-ILD. Mortality in patients with pSS-ILD. Out of 188 articles, seven met the inclusion criteria. The meta-analysis estimated a 5-year survival rate of 82% (73%-91%). Mortality-related factors estimated by the meta-analysis included older age (HRs = 1.06, 95% CI 1.03–1.09, *P* < 0.0001), history of smoking (HRs = 3.44, 95% CI 2.14–5.53, *P* < 0.00001), anti-SSA antibody positivity (HRs = 0.41, 95% CI 0.20–0.85, *P* = 0.02), anti-SSB antibody positivity (HRs = 0.42, 95% CI 0.18–0.98, *P* = 0.04), reduced forced vital capacity (FVC; HRs = 0.96, 95% CI 0.95–0.98, *P* < 0.0001), reduced 6-min walk distance (6MWD; HRs = 0.99, 95% CI 0.99–1.00, *P* = 0.0008), presence of a reticular abnormality (HRs = 3.03, 95% CI 1.54–5.95, *P* = 0.001), and decreased arterial partial pressure of oxygen (PaO_2_) levels (HRs = 0.99, 95% CI 0.97–1.00, *P* = 0.04). The 5-year survival rate for pSS-ILD is 82%. Older age, history of smoking, anti-SSA antibody negativity, anti-SSB antibody negativity, reduced FVC, reduced 6MWD, presence of a reticular abnormality, and decreased PaO_2_ levels increase the mortality risk in pSS-ILD.

**Supplementary Information:**

The online version contains supplementary material available at 10.1007/s10067-024-07191-0.

## Introduction

Primary Sjögren’s syndrome (pSS) is an autoimmune disorder hallmarked by sicca symptoms including keratoconjunctivitis sicca and xerostomia [1]. Complications such as interstitial lung disease (ILD) in pSS patients exacerbate the clinical burden, intensifying morbidity and potentially affecting survival [2]. Despite the clinical importance, the consensus on factors influencing mortality in pSS patients with ILD (pSS-ILD) is lacking. This study aims to employ a meta-analytic methodology to integrate the data and critically appraise the existing evidence, identifying robust determinants of mortality in the pSS-ILD population.

## Methods

### Search strategy

This investigation was rigorously structured in accordance with the Preferred Reporting Items for Systematic Reviews and Meta-Analyses (PRISMA) guidelines to ensure a robust meta-analytical approach [3]. A comprehensive and systematic search was executed across an array of databases, including MEDLINE, EMBASE, Web of Science, Cochrane Library, China National Knowledge Infrastructure (CNKI), and Wanfang Data. The search was delineated with a cutoff date of November 22, 2023, to capture the most recent and relevant studies.

The search strategy for each database was meticulously crafted to optimize the retrieval of pertinent studies. For instance, the search strategy employed in PubMed was articulated as follows: (((((((((prognosis) OR (prognostic factor*)) OR (prognostic variable*)) OR (relevant factor*)) OR (relevant variable*)) OR (related factor*)) OR (related variable*)) OR (contributing factor)) AND (((((Interstitial lung disease*) OR (Lung Disease*, Interstitial)) OR (Pneumoni*, Interstitial)) OR (Interstitial Pneumoni*)) OR (Diffuse Parenchymal Lung Diseases))) AND ((((Sjögren* Syndrome) OR (Syndrome, Sjögren*)) OR (Sicca Syndrome)) OR (Syndrome, Sicca)). The detailed search strategy delineated in Supplementary Table S1.

To enhance the validity of the search results, the database inquiries were independently conducted by two researchers, ensuring the reliability and reproducibility of the search process.

### Study selection

Inclusion criteria are as follows: (i) studies that investigated cohorts with pSS-ILD, confirming pSS diagnoses in accordance with the American-European Consensus Group (AECG) criteria of 2002 [4] or the American College of Rheumatology/European League Against Rheumatism (ACR/EULAR) criteria of 2016 [5]; (ii) those that provided prognostic data pertinent to pSS-ILD or enabled the derivation of essential prognostic data. Studies were excluded based on the following criteria: (i) those not focusing on mortality as an endpoint; (ii) redundant publications, including duplicates, conference abstracts, and dissertations; (iii) reports containing data that were either not credible or incomplete; (iv) researches where raw data were not convertible into a meta-analyzable format, such as enumeration data not transformable into hazard ratios (HR).

### Data extraction and quality assessment

In this systematic review, data extraction was independently and meticulously conducted by two authors, with a subsequent verification process undertaken by two senior reviewers, ensuring the robustness of the methodological framework. The extracted variables included the title, lead author’s name, year of publication, geographic location of the study, study design, cohort size of patients with pSS-ILD, mean age of participants, diagnostic criteria utilized for pSS, and data pertinent to prognosis. The quality of the included studies was appraised using the Newcastle–Ottawa Scale (NOS) [6], with studies achieving a score of 7 or above deemed to be of high quality. A consensus on the quality assessment of studies was reached through a structured discussions between the reviewers.

### Statistical analysis

In this meta-analysis, we sought to elucidate the 5-year survival rates and delineate mortality-associated determinants as reported in the extant literature. Utilizing Review Manager software (version 5.4.1), we computed the 5-year survival rates and their standard errors employing the statistical expressions $P={~}^{X}\!\left/ \!{~}_{N}\right.\times 100\%$ and $SE\left(P\right)=\sqrt{{~}^{P\left(1-P\right)}\!\left/ \!{~}_{N}\right.}$, where ‘P’ is the 5-year survival rates, ‘X’ represents the count of cases still alive after 5 years of follow-up, and ‘N’ denotes the total number of cases. To amalgamate the data, we opted for either fixed-effect or random-effects models to estimate pooled 5-year survival rates, Hazard Ratios (HRs), Risk Ratios (RRs), and their respective 95% Confidence Intervals (95%CIs), thus quantifying the effect size of various factors related to mortality.

The results derived from the random-effects model were presented exclusively in scenarios where substantial heterogeneity is observed among the studies (I^2^ > 50%). In such cases, sensitivity analyses and essential discussion were employed to explore and elucidate the sources of this heterogeneity. Conversely, in the absence of significant heterogeneity, the results from the fixed-effect model were reported. To facilitate a clear and intuitive depiction of the data, forest plots were utilized, displaying the outcomes of individual studies alongside the aggregated estimates. The potential for publication bias was evaluated by Egger’s test if 5 or more studies were available for meta-analysis [7]. A P-value of less than 0.05 was considered to denote statistical significance [8]. The review was not registered.

## Results

### Study characteristics

Our initial search yielded 259 articles, from which 13 duplicates were removed. Subsequent exclusions of conference abstracts, dissertations, reviews, and articles meeting other exclusion criteria resulted in a preliminary selection of 11 studies. Further scrutiny led to the exclusion of an additional 4 studies due to data implausibility or incompleteness, culminating in 7 studies passing the quality assessment and being eligible for the meta-analysis. Among these, 5 reported on the 5-year survival rate in pSS-ILD, and 6 provided insights into mortality-associated factors. The methodical process of study selection is depicted in Flow diagram S1. The baselines characteristics that are included in the study are illustrated in Table 1. For an in-depth view of the quality assessment, readers are directed to the comprehensive table provided in Supplementary Table S2.

**Table 1:** Characteristics of the included studies for the primary Sjögren’s syndrome concomitant with interstitial lung disease (pSS-ILD)

Title	Authors, year and reference	Country	Study design	No. patients	Age^a^	pSS diagnostic criteria	NOS score
Blood KL-6 predicts prognosis in primary Sjögren’s syndrome-associated interstitial lung disease [9]	Kim et al., 2022	South Korea	Retrospective cohort study	46	59.4 ± 10.6	2016 ACR/EULAR	9
Prognostic factors for primary Sjögren’s syndrome-associated interstitial lung diseases [10]	Kamiya et al., 2019	Japan	Retrospective cohort study	99	68 (36–87)	2002 AECG	9
Characteristics and mortality in primary Sjögren syndrome–related interstitial lung disease [11]	Gao et al., 2021	China	Retrospective case–control study	178	61.59 ± 11.69	2002 AECG	8
Clinical characteristics and outcomes in patients with primary Sjogren’s syndrome-associated interstitial lung disease [12]	Alhamad et al., 2021	Saudi Arabia	Retrospective cohort study	84	60.5 ± 13.0	Minor Salivary Gland Biopsy	8
Prognostic Factors in Interstitial Lung Disease Associated with Primary Sjögren’s Syndrome: A Retrospective Analysis of 33 Pathologically–Proven Cases [13]	Enomoto et al., 2013	Japan	Retrospective cohort study	33	66 (62–71)	2002 AECG	9
Risk factors for progression and prognosis of primary Sjögren’s syndrome-associated interstitial lung disease in a Chinese population [14]	Xu et al., 2020	China	Retrospective cohort study	113	61.61 ± 10.99	2002 AECG,2016 ACR/EULAR	9
Prevalence, risk factors, and prognosis of interstitial lung disease in a large cohort of Chinese primary Sjögren syndrome patients [15]	Gao et al., 2018	China	Retrospective case–control study	165	61.25 ± 9.79	2016 ACR/EULAR	8

Abbreviations: *AECG* American-European Consensus, *ACR/EULAR* American College of Rheumatology/European League Against Rheumatism, *NOS* Newcastle–Ottawa Scale

^a^Values are presented as median (range) or mean ± SD

### 5-Year survival rates in patients with pSS-ILD

**Fig. 1:**
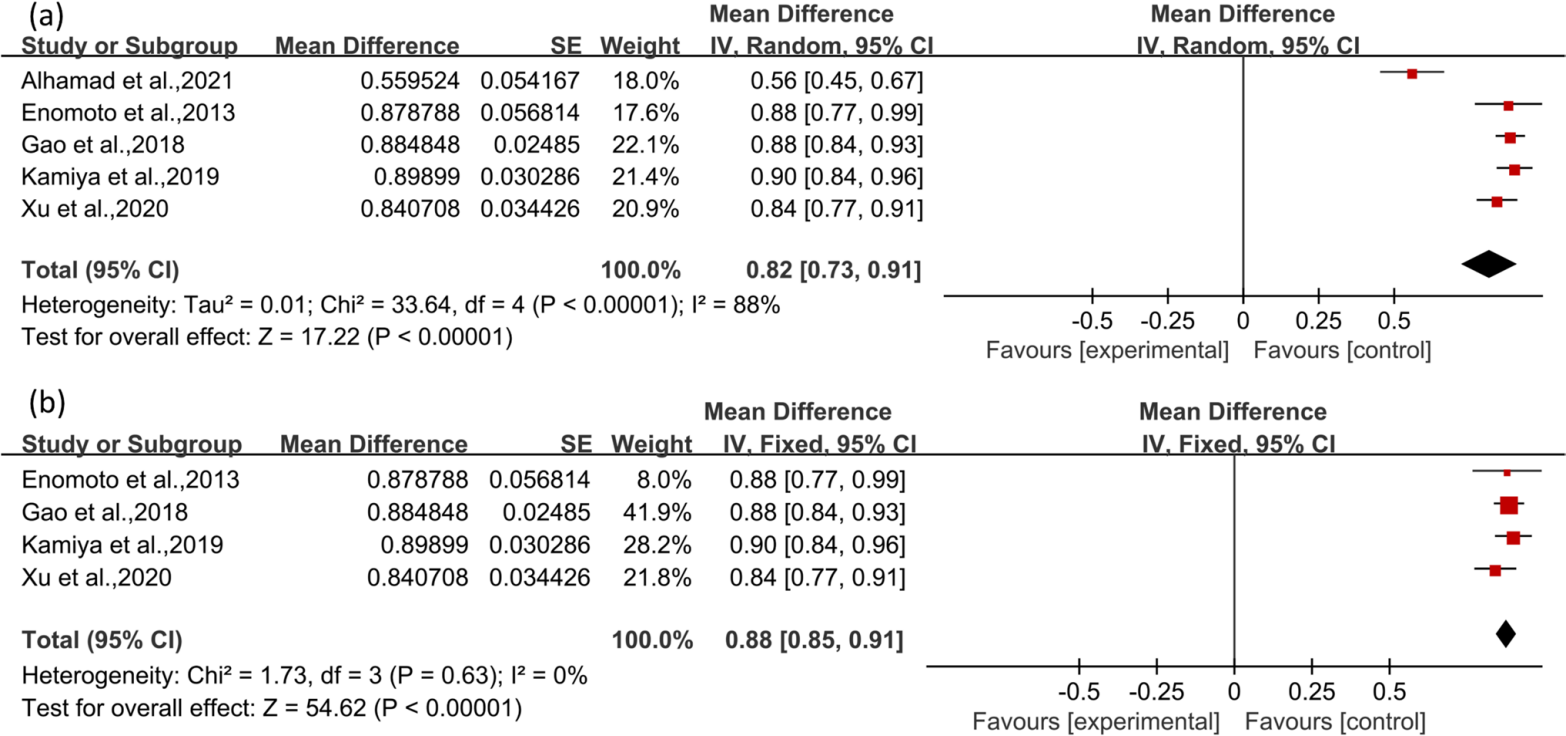
Forest plot (**a**) presenting the 5-year survival rate of patients with pSS-ILD, and (**b**) after sensitivity analysis

We conducted a meta-analysis to estimate the 5-year survival rate in patients with pSS-ILD and depicted forest plot, as shown in Fig. 1a. Given the considerable heterogeneity among the studies (I^2^ = 88%), a random-effects model was utilized. The analysis encompassed data from 494 patients, of whom 406 were reported to be alive at the 5-year follow-up. The aggregated 5-year survival rate was calculated to be 82% (95% CI: 73%-91%). Additionally, the P-value of Egger’s test was 0.09 indicating that there was no evidence of publication bias. A sensitivity analysis, which involved excluding the study conducted by Alhamad et al., led to the elimination of heterogeneity (I^2^ = 0%). This allowed for the adoption of a fixed-effect model, revealing an adjusted pooled 5-year survival rate of 88% (95% CI: 85%-91%), as presented in Fig. 1b.

### Mortality-related factors in patients with pSS-ILD

**Fig. 2:**
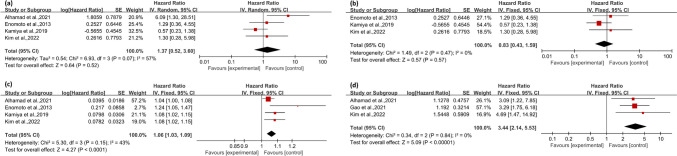
Forest plot illustrating the factors possibly related to mortality (based on patient demographics and baseline characteristics) in patients with pSS-ILD, including male gender (**a** and **b**), age (**c**), and smoking history (**d**)

**Fig. 3:**
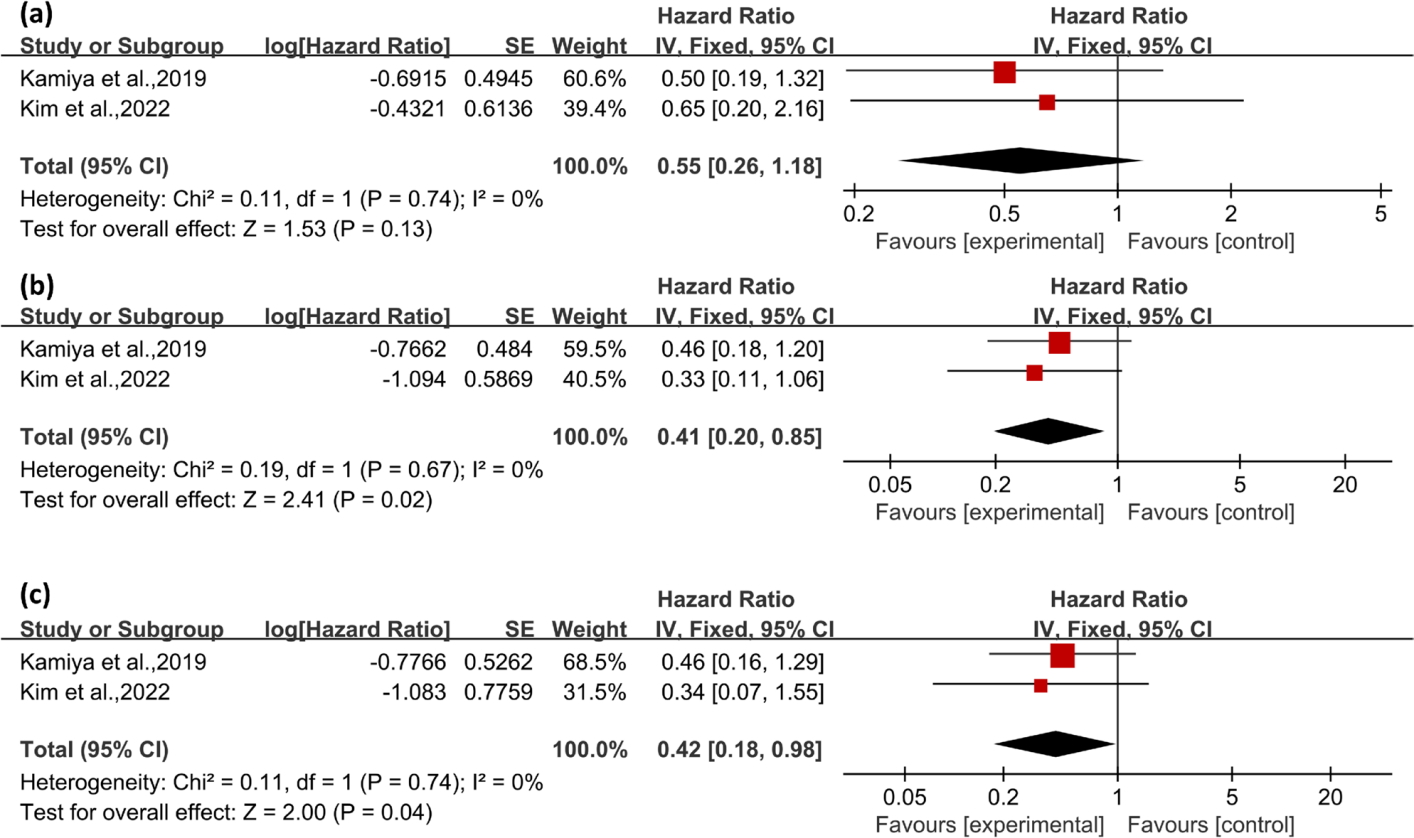
Forest plot depicting the serological parameters possibly associated with mortality in patients with pSS-ILD, including positivity for antinuclear antibodies (**a**), anti-SSA antibodies (**b**), and anti-SSB antibodies (**c**)

**Fig. 4:**
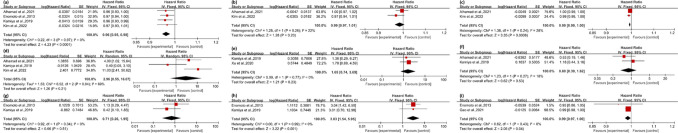
Forest plot demonstrating the pulmonary function variables and HRCT patterns possibly associated with mortality in patients with pSS-ILD, including reductions in forced vital capacity (**a**), diffusion capacity for carbon monoxide of the lung (**b**), and the six-minute walk distance (**c**), as well as HRCT patterns, including the presence of usual interstitial pneumonia (**d**), possible UIP patterns (**e**), non-specific interstitial pneumonia patterns (**f**), honeycombing (**g**), reticular abnormalities (**h**), and a decrease in arterial partial pressure of oxygen (**i**)

A meta-analysis was conducted to identify factors associated with mortality in patients with pSS-ILD, and the results were illustrated using forest plots. The endpoint for each study included in this analysis was mortality. The baseline characteristics and personal history of patients, including male gender, age, and smoking history, are depicted in Fig. 2. Serological parameters related to the disease, such as positivity for antinuclear antibodies (ANA), anti-SSA antibodies, and anti-SSB antibodies, are detailed in Fig. 3. A decrease in PaO_2_, Pulmonary function variables, including reductions in FVC, diffusion capacity for carbon monoxide of the lung (DLCO), and the six-minute walk distance (6MWD), as well as HRCT patterns, including the presence of usual interstitial pneumonia (UIP), possible UIP patterns, non-specific interstitial pneumonia (NSIP) patterns, honeycombing, and reticular abnormalities, are presented in Fig. 4. The aggregated findings indicate that older age, a history of smoking, positive anti-SSA and anti-SSB antibodies, reduced FVC, shortened 6MWD, the presence of a reticular abnormality, and decreased PaO_2_ are risk factors associated with increased mortality in pSS-ILD.

In an analysis designed to elucidate the impact of patient baseline characteristics and personal history on the prognosis of those with Primary Sjögren’s Syndrome with Interstitial Lung Disease (pSS-ILD), we analyzed factors such as male gender, age, and smoking history. The synthesized data suggest that gender does not significantly influence mortality in pSS-ILD, with an HR of 1.37 (95%CI, 0.52 to 3.60; *P* = 0.52) and notable heterogeneity (I^2^ > 50%) (Fig. 2a). A sensitivity analysis, which excluded the study conducted by Alhamad et al., yielded a negligible heterogeneity (I^2^ = 0%), and the HR adjusted to 0.83 (95% CI, 0.43 to 1.59; *P* = 0.57), supporting the absence of a gender-mortality relationship (Fig. 2b). Conversely, age was significantly correlated with mortality in pSS-ILD, with an HR of 1.06 (95% CI, 1.03 to 1.09; *P* < 0.0001), and exhibited low heterogeneity (I^2^ < 50%) (Fig. 2c). Similarly, a history of smoking was significantly associated with increased mortality, with an HR of 3.44 (95% CI, 2.14 to 5.53; *P* < 0.00001) and low heterogeneity (I^2^ < 50%) (Fig. 2d).

In our analysis of serological parameters in patients, we evaluated ANA, anti-SSA antibodies and anti-SSB antibodies with mortality in pSS-ILD. Our synthesized findings suggest no significant association between ANA positivity and mortality in pSS-ILD, with an HR of 0.55 (95%CI 0.26–1.18, *P* = 0.13) and low heterogeneity (I^2^ < 50%) (Fig. 3a). However, the presence of anti-SSA antibodies was found to be significantly associated with mortality in pSS-ILD, with an HR of 0.41 (95% CI 0.20–0.85, *P* = 0.02) and low heterogeneity (I^2^ < 50%) (Fig. 3b). Similarly, the presence of anti-SSB antibodies showed a significant association with mortality in pSS-ILD, with an HR of 0.42 (95% CI 0.18–0.98, *P* = 0.04) and low heterogeneity (I^2^ < 50%) (Fig. 3c).

In our comprehensive analysis of pulmonary function-related variables in patients with pSS-ILD, we observed several associations with mortality. A decrease in FVC was significantly associated with increased mortality (HR = 0.96, 95% CI 0.95–0.98, *P* < 0.0001) (Fig. 4a), with low heterogeneity (I^2^ < 50%). In contrast, a reduction in DLCO did not significantly correlate with mortality (HR = 0.99, 95% CI 0.97–1.01, *P* = 0.20) (Fig. 4b), with low heterogeneity (I^2^ < 50%). Similarly, a decrease in 6MWD was found to be significantly associated with mortality (HR = 0.99, 95% CI 0.99–1.00, *P* = 0.0008) (Fig. 4c), with low heterogeneity (I^2^ < 50%). The UIP pattern results (Fig. 4d) showed no significant correlation with mortality (HR = 2.96, 95% CI 0.55–16.07, *P* = 0.21), with high heterogeneity (I^2^ > 50%), therefore a random-effects model was applied. The possible UIP analysis also revealed no significant association with mortality (HR = 1.65, 95% CI 0.74–3.69, *P* = 0.23) (Fig. 4e), with low heterogeneity (I^2^ < 50%). Furthermore, the pooled results indicated that NSIP was not significantly associated with mortality (HR = 0.80, 95% CI 0.39–1.62, *P* = 0.53) (Fig. 4f), with low heterogeneity (I^2^ < 50%). Honeycombing was also not significantly correlated with mortality (HR = 0.71, 95% CI 0.26–1.95, *P* = 0.51) (Fig. 4g), with low heterogeneity (I^2^ < 50%). However, the presence of reticular abnormalities was significantly associated with an increased risk of mortality (HR = 3.03, 95% CI 1.54–5.95, *P* = 0.001) (Fig. 4h), with low heterogeneity (I^2^ < 50%). Finally, a decrease in PaO_2_ was significantly associated with mortality (HR = 0.99, 95% CI 0.97–1.00, *P* = 0.04) (Fig. 4i), with low heterogeneity (I^2^ < 50%).

## Discussion

In this meta-analysis, we aimed to identify factors associated with the 5-year survival rate and mortality in patients with pSS-ILD. Following the PRISMA guidelines, we systematically searched major platforms and databases. The NOS was utilized to assess the quality of the included studies. We extracted study characteristics and effect estimates for pooled analysis. Our findings indicate that the 5-year survival rate for pSS-ILD is 82%. Factors such as older age, history of smoking, reduced FVC, decreased 6MWD, the presence of reticular abnormalities, and lower PaO_2_ were associated with an increased risk of mortality in pSS-ILD.

In our meta-analysis examining the 5-year survival rates, we encountered significant heterogeneity. The exclusion of the study conducted by Alhamad et al. [12]resulted in a nullification of this heterogeneity (I^2^ = 0%). This phenomenon may be explicated by healthcare disparities, wherein the most severe cases of pSS-ILD are referred to “the ILD and PH Centre at King Saud University Medical City”, resulting in low survival rates. There may therefore be an institutional bias in this study, as mentioned by its authors in the discussion section [12].

In the meta-analysis exploring the impact of male gender on survival, significant heterogeneity was noted. This heterogeneity was eliminated (I^2^ = 0%) after the removal of the study by Alhamad et al. [12]. It is postulated that the source of this heterogeneity may be attributable to differences in ethnic backgrounds, as the cohorts in the remaining studies predominantly consisted of East Asian individuals, whereas Alhamad et al.’s cohort was primarily composed of Arab individuals. Consequently, within East Asian populations, gender does not appear to be associated with mortality in pSS-ILD. However, in Arab populations, we inferred that male gender may be conjectured to be a relevant factor associated with mortality in pSS-ILD. This speculation warrants further investigation to substantiate this observation.

A review by Cho et al. [16] has identified aging as one of the risk factors for interstitial lung diseases, particularly idiopathic pulmonary fibrosis (IPF). At the cellular level, there is a profound link between aging and the pathophysiology of IPF, as evidenced by the increased expression of senescence biomarkers. Aging may lead to cellular growth arrest and reduced replicative capacity, further contributing to pulmonary fibrosis by impeding the regeneration of alveolar progenitor cells and promoting the generation of pro-fibrotic cells [17]. Studies by Zhang et al. [18] and Roca et al. [19] also indicate that age is a risk factor for the progression of pSS-ILD, corroborating the findings of our meta-analysis. When examining the history of smoking, the results suggest a significant association with mortality in pSS-ILD (*P* < 0.001). Cigarette smoke may affect abnormal activation of alveolar epithelial cells, thereby contributing to IPF [20]. Furthermore, studies have proposed that aging and smoking may interact with certain environmental factors to induce an aberrant epithelial secretion pattern and increase resistance to apoptosis in myofibroblasts. This could lead to the accumulation of extracellular matrix and the development of pulmonary fibrosis, heightening susceptibility to IPF [21].

Intriguingly, despite studies by Kim et al. [9] and Kamiya et al. [10] indicating that the positive anti-SSA and anti-SSB antibodies does not reduce the mortality risk in patients with pSS-ILD, a pooled analysis reveals a significant association between antibody positivity and a decreased mortality risk in pSS-ILD (Fig. 3b and c). This suggests that the positive anti-SSA and anti-SSB antibodies may confer a protective effect, whereas their negative result could be associated with an increased risk of death in pSS-ILD. The reasons for this result might be explained from the following aspects: ①From a statistical perspective, one role of meta-analysis is to increase the sample size of the original studies, thereby improving statistical precision. The fact that I2 = 0 indicates excellent consistency among the studies, leading to narrower confidence intervals for the combined results, which can explain why they became statistically significant. ②From the perspective of disease diagnosis and treatment, the presence of anti-SSA and anti-SSB antibodies helps confirm the diagnosis of pSS, leading to earlier and more proactive treatment for these patients, which may have improved long-term outcomes, including reducing mortality. Of course, we hope that future studies will provide further evidence supporting this observation from both mechanistic and clinical aspects.

While the study by Kim et al. [9] suggests that a reduced FVC does not increase mortality risk in pSS-ILD, a meta-analysis of FVC data indicates a significant correlation with mortality in pSS-ILD (*P* < 0.0001), signifying that a decrease in FVC is associated with an elevated risk of death. The study by Ley et al. [22] has demonstrated that FVC is the most sensitive parameter for assessing the clinical course in patients with IPF. Similarly, the study by He et al. [23] indicates that FVC is a risk factor for the progression of pSS-ILD. A reduction in 6MWD is also associated with increased mortality in pSS-ILD, aligning with previous research [24, 25]. These pulmonary function parameters warrant particular attention in clinical practice.

In the study by Kamiya et al., the presence of reticular abnormalities was not associated with increased mortality in patients with pSS-ILD. However, the pooled analysis indicates that reticular abnormalities may indeed elevate the risk of mortality in pSS-ILD. reticular abnormalities are often considered a subtle feature of fibrosis in ILD and have been identified as a risk factor for disease progression in previously published clinical practice guidelines [26]. This is corroborated by the findings of He et al. [23], which also suggest that the emergence of reticular abnormalities poses a risk for the progression of pSS-ILD. While the study by Enomoto et al. reported that a reduction in PaO_2_ does not increase mortality risk in pSS-ILD, the aggregated analysis results reveal a significant association, indicating that a decrease in PaO_2_ is associated with an elevated risk of mortality in pSS-ILD. In a study by Ito et al. [27], which included a sample of 33 cases, it was shown that a decrease in PaO2 (*p* = 0.02) was independently associated with survival in pSS, which side-steps the credibility of this finding.

This investigation boasts several advantages and limitations. Foremost, it not only fills a void in the extant body of research but also holds significant implications for clinical practice. The findings provide physicians with a foundation for identifying patients at heightened risk and executing early interventions, thereby potentially enhancing patient prognoses. Additionally, the high quality of the included literature and the low heterogeneity across most groups bolster the reliability and generalizability of the study outcomes, mitigating the impact of errors. Nonetheless, certain limitations are present within our study. The number of studies incorporated into the meta-analysis is relatively modest, which may affect the stability of the analysis. Furthermore, a small subset of the literature demonstrates considerable heterogeneity, which could introduce bias into the analytical results. Consequently, future research should aim to increase the volume and sample size of the literature included and improve the quality of data to elevate the credibility and dependability of the findings.

In conclusion, this study elucidates key determinants of mortality in patients with pSS-ILD, providing clinicians with more precise prognostic indicators and informing subsequent research trajectories. It is our hope that forthcoming studies will continue to reveal critical prognostic indicators and refine therapeutic approaches to substantially benefit survival rates and enhance the quality of life in patients with pSS-ILD.

## Conclusion

In summary, our comprehensive meta-analysis discloses a five-year survival rate of 82% in patients diagnosed with pSS-ILD. We identified several factors linked with elevated mortality risk in pSS-ILD, including older age, history of smoking, negative anti-SSA and anti-SSB antibodies, diminished FVC, reduced 6MWD, the manifestation of a reticular abnormality, and decreased PaO_2_.

## Supplementary Information

Below is the link to the electronic supplementary material.

Supplementary file1 (DOCX 19 KB)

Supplementary file2 (DOCX 32 KB)

Fig. S1 (PNG 281 KB)

Supplementary file3 (TIF 4948 KB)

## Data Availability

Not applicable.
