# Supplementary material for: Meta-analysis of mortality-associated factors in primary Sjögren’s syndrome patients with interstitial lung disease
Source: Clin Rheumatol. 2024 Oct 21;44(1):23–31. doi: 10.1007/s10067-024-07191-0 (PMC11729075; doi:10.1007/s10067-024-07191-0)
Supplement: Supplementary file 1 — Supplementary file1 (DOCX 19 KB) [file 10067_2024_7191_MOESM1_ESM.docx]

**Table S1. The detailed search strategy**

| Search | Query | Result |
| --- | --- | --- |
| #1 | Search: (((((((prognosis) OR (prognostic factor*)) OR (prognostic variable*)) OR (relevant factor*)) OR (relevant variable*)) OR (related factor*)) OR (related variable*)) OR (contributing factor) | 4,446,908 |
| #2 | Search: ((((interstitial lung disease*) OR (Lung Disease*, Interstitial)) OR (Pneumoni*, Interstitial)) OR (Interstitial Pneumoni*)) OR (Diffuse Parenchymal Lung Diseases) | 103,797 |
| #3 | Search: (((Sjogren* Syndrome) OR (Syndrome, Sjogren*)) OR (Sicca Syndrome)) OR (Syndrome, Sicca) | 22,020 |
| #4 | Search: (((((((((prognosis) OR (prognostic factor*)) OR (prognostic variable*)) OR (relevant factor*)) OR (relevant variable*)) OR (related factor*)) OR (related variable*)) OR (contributing factor)) AND (((((interstitial lung disease*) OR (Lung Disease*, Interstitial)) OR (Pneumoni*, Interstitial)) OR (Interstitial Pneumoni*)) OR (Diffuse Parenchymal Lung Diseases))) AND ((((Sjogren* Syndrome) OR (Syndrome, Sjogren*)) OR (Sicca Syndrome)) OR (Syndrome, Sicca)) | 268 |

**Table S2**. **The quality of included studies in meta-analysis based on the Newcastle-Ottawa scale**

Table S2a. Study quality of cohort studies

| Author | Representativeness of the exposed cohort | Selection of the non- exposed cohort | Ascertainment of exposure | Demonstration that outcome of interest was not present at start of study | Comparability of cohorts on the basis of the design or analysis | Assessment of outcome | Was follow-up long enough for outcomes to occur | Adequacy of follow up of cohorts | Total scores |
| --- | --- | --- | --- | --- | --- | --- | --- | --- | --- |
| Kim et al.,2022 | ★ | ★ | ★ | ★ | ★★ | ★ | ★ | ★ | 9 |
| Kamiya et al.,2019 | ★ | ★ | ★ | ★ | ★★ | ★ | ★ | ★ | 9 |
| Alhamad et al.,2021 | ★ | ★ | ★ | ★ | ★★ | ★ | ☆ | ★ | 8 |
| Enomoto et al.,2013 | ★ | ★ | ★ | ★ | ★★ | ★ | ★ | ★ | 9 |
| Xu et al.,2020 | ★ | ★ | ★ | ★ | ★★ | ★ | ★ | ★ | 9 |

Table S2b. Study quality of case-control studies

| Author | Is the case definition adequate? | Representativeness of the cases | Selection of controls | Definition of controls | Comparability of cases and controls on the basis of the design or analysis | Ascertainment of exposure | Same method of ascertainment for cases and controls | Non-response rate | Total scores |
| --- | --- | --- | --- | --- | --- | --- | --- | --- | --- |
| Gao et al.,2021 | ★ | ★ | ★ | ★ | ★ | ★ | ★ | ★ | 8 |
| Gao et al.,2018 | ★ | ★ | ★ | ★ | ★ | ★ | ★ | ★ | 8 |
