# Supplementary figures and images for: Meta-analysis of mortality-associated factors in primary Sjögren’s syndrome patients with interstitial lung disease
Source: Clin Rheumatol. 2024 Oct 21;44(1):23–31. doi: 10.1007/s10067-024-07191-0 (PMC11729075; doi:10.1007/s10067-024-07191-0)

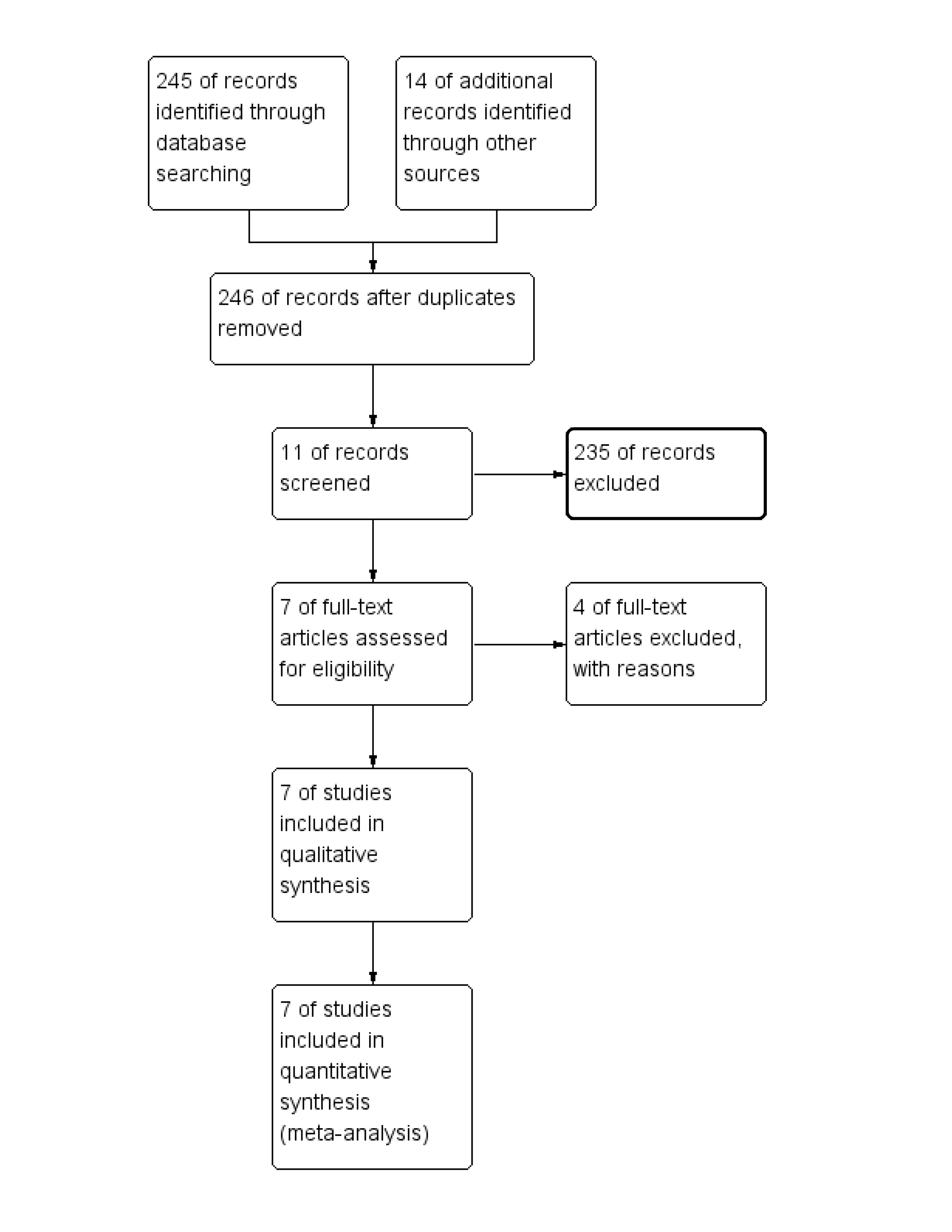

Supplement: Supplementary file 3 — (PNG 281 KB) [file 10067_2024_7191_Fig5_ESM.png]
